# Single‐cell and spatial transcriptomics uncover neoadjuvant chemotherapy‐resistant malignant cells with inhibitory signalling on B cells in gastric cancer

**DOI:** 10.1002/ctm2.70600

**Published:** 2026-02-02

**Authors:** Pei‐Yi Han, Xiang‐Xi Ye, Xiao Yang, Lin Li, Xuan Zhao, Yan‐Fei Shao, Jing Sun, Lu Zang, Ze‐Guang Han, Min‐Hua Zheng

**Affiliations:** ^1^ Division of Gastrointestinal and Colorectals Surgery, Department of General Surgery Ruijin Hospital, Shanghai Jiao Tong University, School of Medicine Shanghai China; ^2^ Shanghai Institute of Minimally Invasive Surgery, Ruijin Hospital, Shanghai Jiao Tong University, School of Medicine Shanghai China; ^3^ Key Laboratory of Systems Biomedicine (Ministry of Education) and State Key Laboratory of Medical Genomics, Shanghai Center for Systems Biomedicine, Shanghai Jiao Tong University Shanghai China; ^4^ Department of General Surgery Zhongshan Hospital, Fudan University Shanghai China; ^5^ Department of Digestive Diseases Huashan Hospital, Fudan University Shanghai China

1

Dear editor,

Patients with advanced gastric cancer (GC) often receive the neoadjuvant therapy with variable responsiveness. Here, we conducted single‐cell RNA sequencing on tumour tissues from GC patients receiving neoadjuvant chemotherapy, integrated with spatial transcriptomics analysis, revealing that resistance to neoadjuvant chemotherapy appears to be driven by SPP1‐CD44 axis, which drives immunosuppressive crosstalk between apoptosis‐resistant undifferentiated malignant cells and B cells.

GC remains one of the leading causes of cancer‐related mortality worldwide.^1^ Neoadjuvant chemotherapy has become a standard treatment modality for these patients with advanced GC but does not always yield satisfactory outcomes.^2^ Activating of anti‐apoptotic signalling pathways,^3^ the immunosuppressive microenvironment tumour‐associated macrophages (TAMs) and regulatory T cells (Tregs) may contribute to the resistance to chemotherapy.^4^, ^5^ Recent studies have shown that activated B cells can enhance anti‐tumour responses by secreting antibodies and cytokines,^6^ but B cells can also be reprogrammed to promote tumour growth and immune evasion.^7^ The underlying molecular and cellular characteristics remain unclear.

In this study, we performed multi‐omics analysis on tumour tissues from five patients (including 3 responders and 2 non‐responders) treated with DOS, SOX and XELOX regimens for 3 or 4 cycles (Table S1), according to the response evaluation criteria in solid tumours (RECIST).^8^ Whole‐exome sequencing revealed that somatic mutations in some genes existed in the residual tumours of all five patients. Interestingly, amongst these genes, ATR, a master regulator of cellular responses to DNA replication stress; ABCB1, an ATP‐dependent drug efflux pump for xenobiotic compounds; and NSD1, a histone methyltransferase, have been reported to be involved in anticancer drug resistance. In addition, the mutations of *EPB41L3*, *TFG*, *TAL2*, *WWTR1* and *ARIH1* were observed in responders, whereas nonsynonymous SNV and frameshift deletion in *CCNB1IP* existed in the non‐responders (Figure 1A,B). COSMIC signature analysis showed that SBS24 and SBS36, which are respectively associated with aflatoxin exposure and defective base excision repair, appeared to have more contribution in responders (Figure 1C). Transcriptomic analysis showed 161 upregulated and 439 downregulated genes in non‐responders (Figure 1D). Interestingly, gene ontology (GO) enrichment analysis revealed suppressed immune response and antibody production in non‐responders (Figure 1E).

**FIGURE 1:**
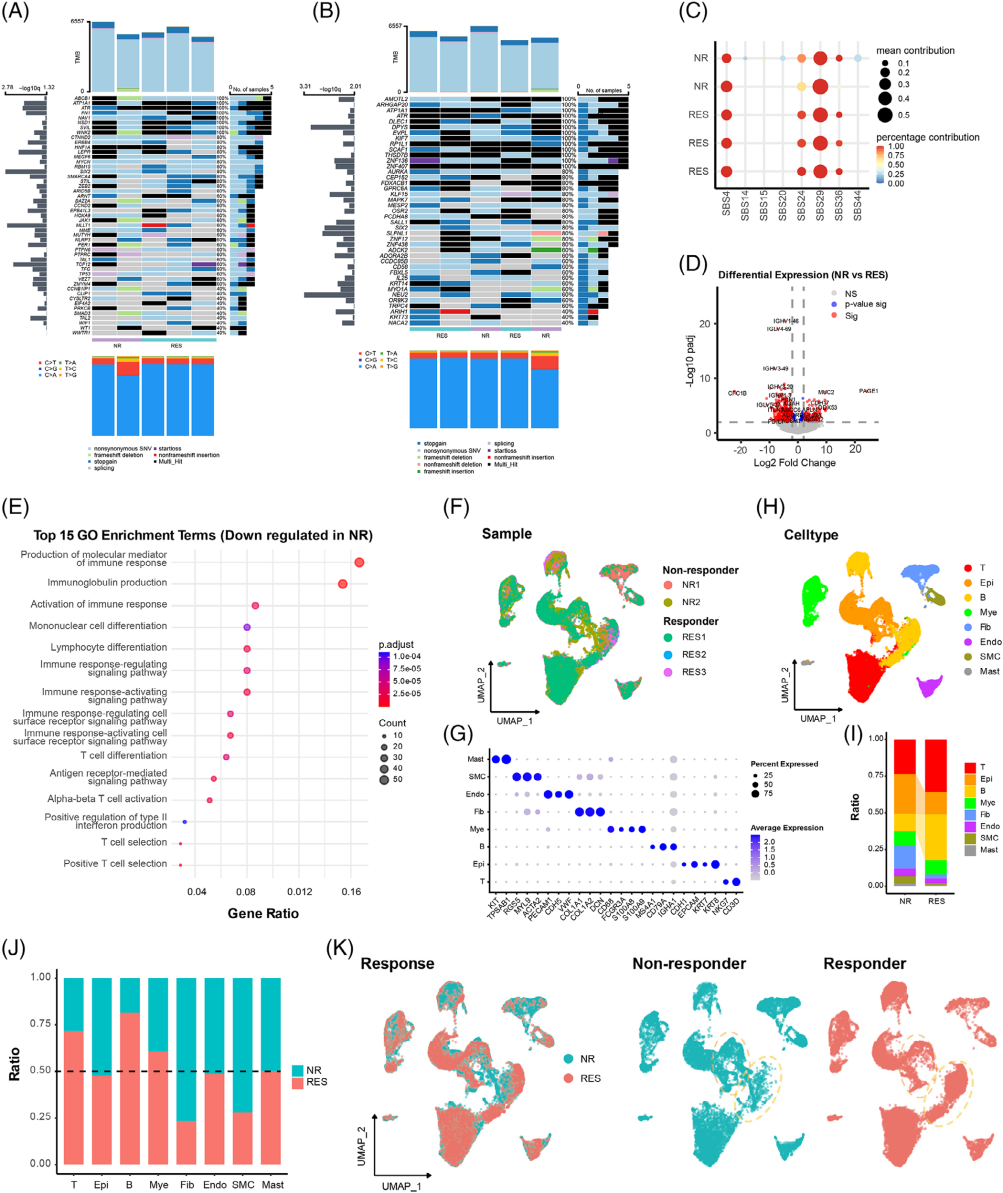
Integrated genomic and cellular landscape distinguishing responders and non‐responders to neoadjuvant chemotherapy in gastric cancer. (A) Waterfall map showing the mutation of known driver genes deposited in COSMIC dataset in each sample. (B) Waterfall map showing the mutation of significant genes via statistically analysis using MutSigCV1 in each sample. (C) Bubble plot demonstrating the contribution of each mutation signature in each sample. (D) Volcano map illustrating differentially expressed genes between the responding and non‐responding group. Blue dots (*p*‐value sig) indicate genes that meet the *p*‐value significance threshold only; red dots (Sig) represent genes that are statistically significant based on both a *p*‐value threshold and a Log2 fold change threshold of ± 2.0. (E) Top 15 pathways significantly enriched in genes down‐regulated in the non‐responding group. (F) uniform manifold approximation and projection (UMAP) plot displaying scRNA‐seq data from tumour tissues of 5 GC patients, including two non‐responders (NR1, NR2) and three responders (RES1, RES2, RES3). (G) Dot plot showing the expression of marker genes (T: NK/T cells, Epi: epithelial cells, B: B cells, Mye: myeloid cells, Fib: fibroblasts, Endo: endothelial cells, SMC: smooth muscle cells, Mast: mast cells). (H) UMAP plot coloured by cell type. (I) Bar plot comparing the ratio of different cell types between non‐responders (NR) and responders (RES). (J) Bar plot showing the ratio of non‐responders (NR) and responders (RES) within each cell type. (K) UMAP plots separated by response group (NR and RES), with yellow dashed circles representing epi (upper) and B cell (lower) clusters.

Furthermore, single‐cell RNA sequencing of 36,910 cells identified 8 major cell types (Figure 1F–H). Amongst these, epithelial cells, fibroblasts and smooth muscle cells were enriched in non‐responders, whereas B cells were reduced, also suggesting weakened adaptive immunity (Figure 1I–K). The inferred copy number variation (CNVs) analysis distinguished 6,610 malignant epithelial cells from 606 normal ones (Figures 2A and S1A,B), revealing seven malignant subclusters (EPI1–7; Epithelial cells [EPI]) (Figure 2B). Significantly, EPI5 was preferentially enriched in non‐responders (Figure 2C,D). The trajectory analysis demonstrated a pronounced directional flow from the EPI5 subpopulation towards other malignant cell subpopulations (Figure 2E). Pseudotime and CytoTRACE analyses positioned EPI5 at the earliest differentiation stage, indicating a progenitor‐like phenotype (Figure 2F,G).

**FIGURE 2:**
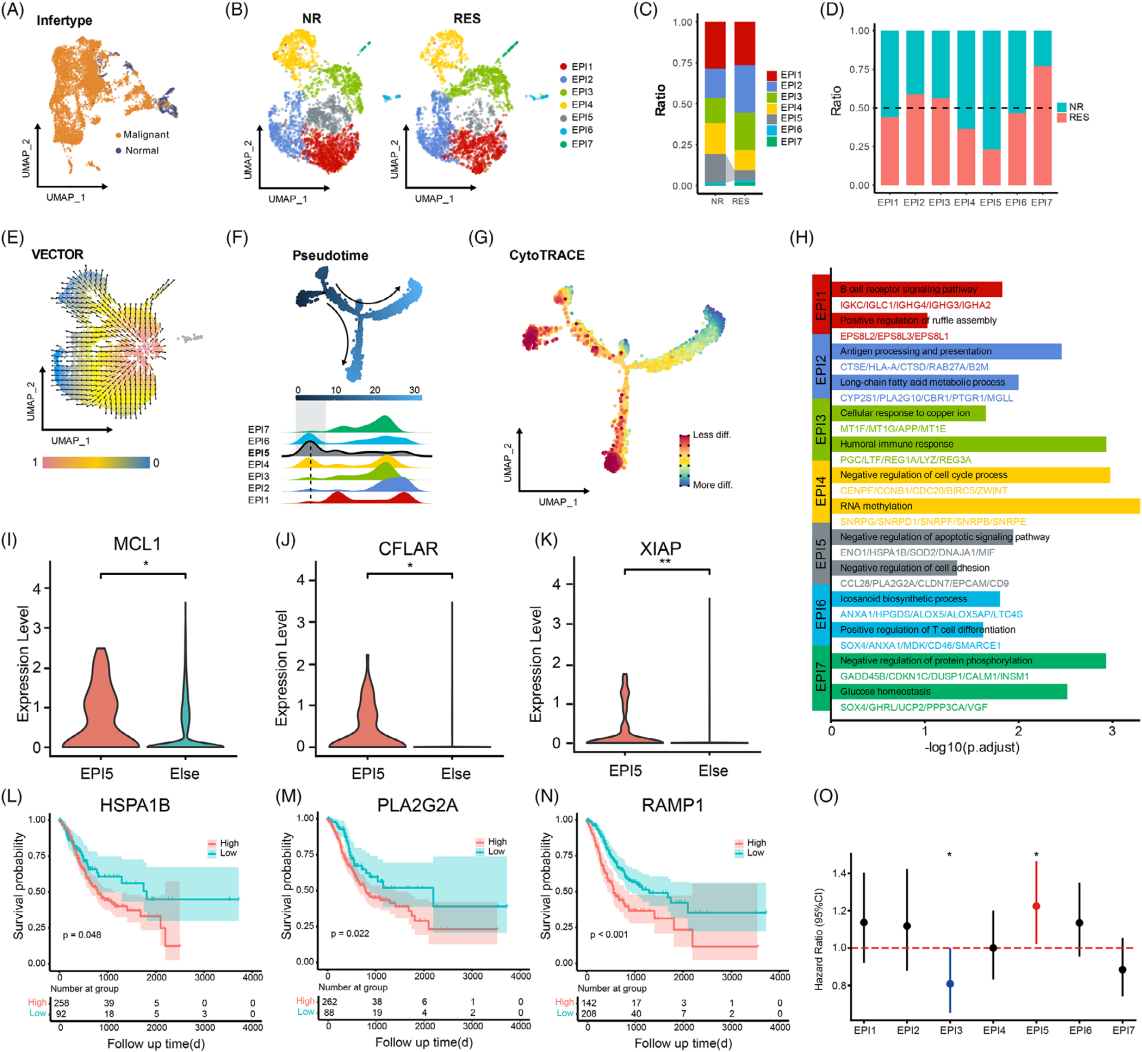
Characteristics and distribution of malignant epithelial cells in gastric cancer patients undergoing neoadjuvant therapy. (A) UMAP plot distinguishing malignant and normal epithelial cells based on inferCNV. (B) UMAP plots showing the distribution of 7 distinct malignant epithelial cell subpopulations (EPI1‐7) in non‐responders (NR) and responders (RES) separately. (C) Bar plot depicting the ratio of each malignant epithelial subpopulation (EPI1‐7) in NR and RES. (D) Bar plot comparing the proportions of NR and RES in each EPI subpopulation. (E) VECTOR trajectory analysis illustrating the pseudotime trajectory of malignant epithelial cells. The background colour mapping represents the quantile polarisation (QP) value for each cell. (F) Monocle pseudotime analysis with the density of each EPI subpopulation along the trajectory. (G) CytoTRACE analysis showing the differentiation states of malignant epithelial cells. (H) Enrichment analysis of highly expressed genes in each EPI subpopulation. (I–K) Expression level of MCL1, CFLAR and XIAP of EPI5. (L–N) Kaplan–Meier survival curves showing the association between high and low expression levels of EPI5 markers (HSPA1B, PLA2G2A and RAMP1) and overall survival in GC patients. (O) Cox multivariate risk regression analysis of different EPI clusters. GC, gastric cancer.

Interestingly, EPI5 exhibited the features of stem‐like, undifferentiated, anti‐apoptotic cells with reduced adhesion (Figure 2H). Specifically, the EPI5 cluster exhibits a specialised anti‐apoptotic phenotype, supported by the upregulation of key survival regulators MCL1, CFLAR and XIAP (Figure 2I–K), alongside gene set enrichment analysis (GSEA) confirmation of the CDKN1A‐mediated survival pathway enrichment (Figure S2A). High expression of EPI5 marker genes (HSPA1B, PLA2G2A and RAMP1) in the TCGA‐STAD cohort correlated with poor overall survival (Figures 2L–N and S2B–D), suggesting their prognostic value. We further leveraged the TCGA data to probe the association of the EPI subset signature with the overall survival of cancer patients. In cox multivariate risk regression analysis, the EPI5 cell group was an independent risk factor for poor prognosis, with statistically significant differences (Figure 2O).

To investigate whether the EPI5 subpopulation interacts with B cells and thereby contributes to the formation of an immunosuppressive tumour microenvironment, we performed cell–cell communication analysis, revealing the ligand–receptor interactions between various malignant epithelial cell subpopulations (EPI1–7) and B cells through MIF, and its receptors CD74, CD44 and CXCR4 (Figure 3A). Notably, the known SPP1‐CD44 axis‐mediated immunosuppressive signalling pathway only existed in interactions between EPI5 cells and B cells. EPI5 acted as a dominant sender of SPP1 signals, whereas B cells and some epithelial subsets served as receivers (Figure 3B,C). To characterise the role of B cells in response to the neoadjuvant therapy, we further extracted B cell information from the scRNA‐seq data of the five patients (Figure 3D). B cells were identified as four subtypes: activated B cells, FKBP11^+^ plasma cells, TXN^+^ plasma cells, and mitochondria‐related B cells (Figure 3E,F). Activated B cells accounted for ∼50% of B cells in responders but only ∼10% in non‐responders, implying that the activated B cells could be suppressed in non‐responders. Furthermore, GSEA showed protein synthesis and T‐cell regulatory pathways were enhanced in activated B cells of responders (Figure 3G), whereas these functions were suppressed in non‐responders. CD44 and CD83 were highly expressed in activated B cells,^9^ supporting their activation potential (Figure 3H). Conversely, both XBP1, a transcription factor responsible for regulating MHC class II genes, and TNFRSF17, a member of the TNF‐receptor superfamily, were highly expressed in plasma cells, but not in activated B cells (Figure 3H). SCENIC analysis revealed the activation of transcription factors HOXA11, GATA2, and JUNB in activated B cells, but not in plasma cells from non‐responders, indicating functional decline (Figure 3I). Interestingly, EPI5 exerted the strongest interaction weight on activated B cells (Figure 3J). Notably, the known immunosuppressive SPP1‐CD44 signalling axis exhibited a significantly stronger intensity between EPI5 and activated B cells compared to its interactions with other B cell subpopulations (Figure 3K). Furthermore, the pathway role analysis revealed that EPI5 functioned as the primary sender of SPP1 signal molecule, whilst the activated B cells served as the main receivers (Figure 3L,M).

**FIGURE 3:**
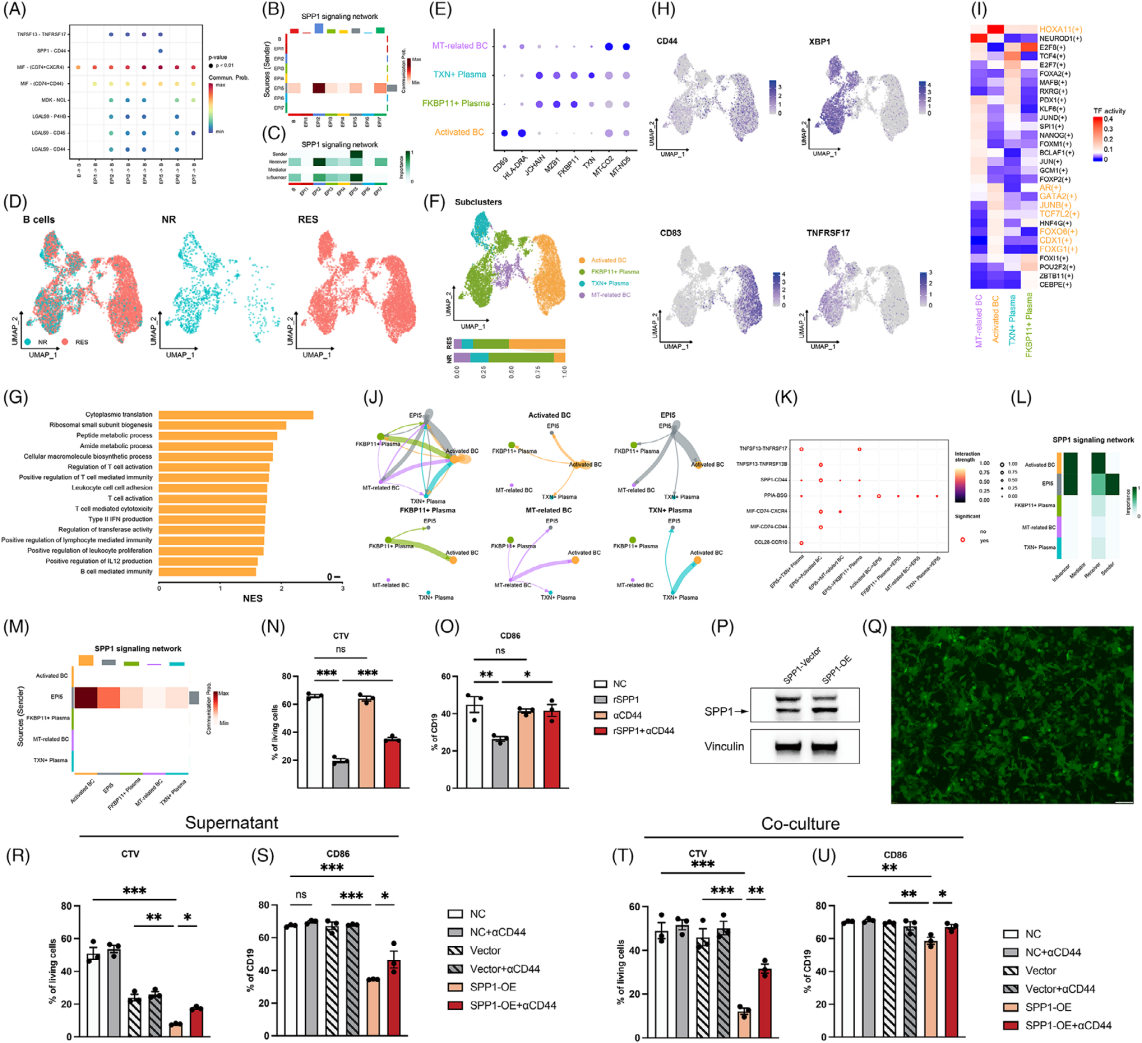
Integrated analysis of malignant epithelial–B cell interactions reveals immunosuppressive signalling underlying therapeutic resistance and poor prognosis in gastric cancer. (A) Dot plot of significant ligand gastric cancer receptor interactions between malignant epithelial cell subpopulations (EPI1‐7) and B cells. (B) Heatmap of the SPP1 signalling network showing communication probabilities between different cell types. (C) Importance and roles of cell types in the SPP1 signalling network. (D) UMAP plots of B cells separated by response group (NR and RES). (E) Dot plot showing the expression of marker genes. (F) UMAP plot coloured by cell type with bar plot of cell composition between respond group. (G) Bar plot showing GSEA results of activated B cells. NES, normalised enrichment score. (H) Feature plot showing the expression of specific genes in B cells. (I) Heat map of transcription factor activity across the distinct cell types. (J) Weights of specific cell–cell interaction. (K) Dot plot of significant ligand–receptor interactions between anti‐apoptotic epithelial cells (EPI5) and B cells. (L,M) Heatmap of the SPP1 signalling network showing the importance (L) and communication probabilities (M) between different cell types. (N) Cell trace violet (CTV) of B cells in different groups. (O) CD86 positive B cell rate in different groups. (P) Western blot of SPP1‐vector and SPP1‐OE MFC tumour cells. (Q) The infection efficiency of SPP1‐OE MFC tumour cells. (R–U) CTV of B cells (R) and CD86 positive B cell rate (U) in different groups.

We also analysed published PRJEB45598 GC single‐cell sequencing data from samples taken before and after the neoadjuvant therapy.^10^ Differential population abundance analysis, performed via Milo algorithm, revealed that the EPI5 cluster was significantly enriched within the NR cohort compared to RES cohort (Figure S3A). Whilst most other epithelial subsets declined following treatment, EPI5 populations remained relatively stable before and after the neoadjuvant therapy (Figure S3B), suggesting an intrinsic drug‐resistant phenotype. Simultaneously, a significant reduction in B cell frequency was observed post‐treatment specifically in the NR group (Figure S3C), further supporting the hypothesis that the reduced B cell abundance is potentially linked to the presence of EPI5 characteristics.

To comprehensively validate the functional impact of the SPP1‐CD44 axis on B cells, we performed a series of in vitro experiments. As expected, treatment with recombinant SPP1 significantly attenuated primary B cell proliferation and activation, but the inhibitory effect was effectively reversed by blocking with an anti‐CD44 antibody (Figure 3N,O). Subsequently, we established SPP1‐overexpressing (OE) MFC cell line, confirmed via fluorescence microscopy and Western blotting (Figure 3P,Q). Consistent with the recombinant SPP1, the supernatant from SPP1‐OE MFCs significantly inhibited B cell functions (Figure 3R,S). A similar phenotype was also observed in direct co‐culture between SPP1‐OE MFC and primary B cells (Figure 3T,U). Crucially, CD44 blockade reversed the inhibitory effects in both experimental settings, collectively substantiating the notion that the SPP1‐CD44 axis mediates B cell suppression.

To further confirm the proposed relationship between EPI5 and B cells, we performed spatial transcriptomics analysis on the sample from a non‐responder (GSE251950). By comparing the pathological slices and the spatial distribution patterns, we found that the resistant‐related malignant epithelial cells (EPI5) were predominantly concentrated in the core of the different microtumour foci, whereas the activated B cells were mainly situated in stroma‐rich areas (Figure 4A). Colocalisation analysis indicated that certain resistant‐related epithelial cells and activated B cells were aggregated within the same regions (Figures 4B and S4A). The homologous cell network analysis was conducted to qualify the abundance of activated B cells and resistant‐related epithelial cells, respectively (Figure 4C). Interestingly, the distribution degree of resistant‐related epithelial cells was negatively associated with that of activated B cells within microtumour foci (Figures 4D and S4B). Immunofluorescence staining of non‐responder tissues corroborated these spatial patterns: CD44^+^ B cells and SPP1^+^ tumour cells aggregated within the same regions yet lacked direct colocalisation (Figure S4C). We also conducted COMMOT cell signalling flow analysis, which revealed extensive areas of SPP1 signal transmission on the tissue section (Figure 4E). In this context, SPP1 signal flows from regions enriched with the resistant‐related epithelial cells to those enriched with activated B cells (Figure 4F), consistent with the inference derived from scRNA‐seq data.

**FIGURE 4:**
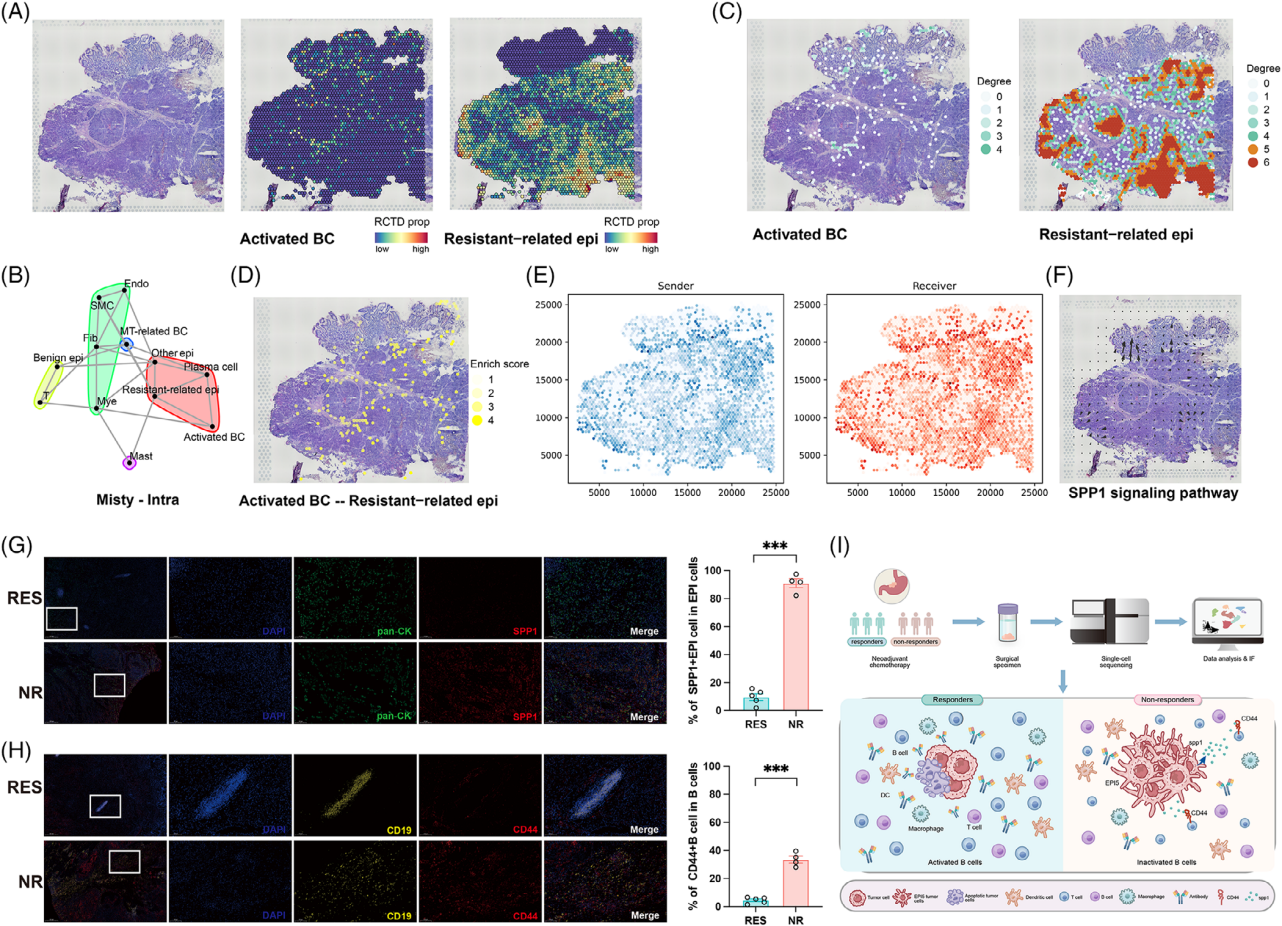
Spatial transcriptomics and histological validation of colocalisation and signal flow between resistant epithelial cells and B cells. (A) Feature plot demonstrating the distribution and colocalisation of activated B cells and resistant malignant cells within each spot. (B) Cell type colocalisation network. (C) Homotypic cell networks showing the intensity of enrichment areas of activated B cells and resistant malignant cells based on spatial transcriptomics analysis. (D) Heterotypic cell networks showing the areas of possible interaction between activated B cells and resistant malignant cells. (E) Sending and receiving of SPP1 signalling in the pathological section. (F) Flow direction of SPP1 signalling on the pathological section. (G) Typical image showing SPP1‐positive epithelial cells in epithelial cells of GC tissues after neoadjuvant chemotherapy, as determined by IF staining (left), and statistical analysis of these SPP1‐positive epithelial cells between NR (*n* = 4) and RES (*n* = 5) groups (right). (H) Typical image showing CD44‐positive B cells in GC tissues after neoadjuvant chemotherapy, as determined by IF staining (left), and statistical analysis of these CD44‐positive B cells in GC tissues from NR (*n* = 4) and RES (*n* = 5) groups (right). (I) Graphical abstract. GC, gastric cancer.

Finally, we performed immunofluorescence staining on postoperative specimens from 9 GC patients (Table S2). Tumour tissues from non‐responders exhibited a significant increase in the number of SPP1‐positive epithelial cells (Figure 4G). Concurrently, the proportion of CD44+ B cells amongst the total B cell populations was also significantly higher in these tissues (Figure 4H).

In conclusion, our study shows that resistance to neoadjuvant chemotherapy in GC appears to be driven by SPP1‐mediated immunosuppressive crosstalk between apoptosis‐resistant undifferentiated malignant cells and B cells. Targeting the SPP1‐CD44 axis or EPI5 subpopulation may represent a promising strategy to overcome treatment resistance and improve patient outcomes.

## AUTHOR CONTRIBUTIONS

Study conception and design was the responsibility of Min‐Hua Zheng, Jing Sun, Lu Zang, Pei‐Yi Han and Ze‐Guang Han. Experiments and data collection were performed by Pei‐Yi Han, Xiang‐Xi Ye, Yan‐Fei Shao, Xuan Zhao, Jing Sun and Lu Zang. Computation and statistical analysis was carried out by Pei‐Yi Han, Xiang‐Xi Ye, Yan‐Fei Shao, Jing Sun and Lin Li. Data interpretation and biological analysis was performed by Pei‐Yi Han, Xiang‐Xi Ye, Yan‐Fei Shao, Lu Zang and Lin Li. The manuscript was written by Pei‐Yi Han, Xiang‐Xi Ye and Xiao Yang, and was critically revised by Ze‐Guang Han. Administration was carried out by Ze‐Guang Han, Jing Sun and Min‐Hua Zheng.

## CONFLICT OF INTEREST STATEMENT

The authors declare no conflicts of interest.

## ETHICS APPROVAL AND CONSENT TO PARTICIPATE

All protocols for this study were reviewed and approved by Ruijin Hospital Institutional Ethics Committee.

## Supporting information



Supporting information

Supporting information

Supporting information

Supporting information

Supporting information

Supporting information

Supporting information

Supporting information

## Data Availability

The datasets used and/or analysed during the current study are available from the corresponding authors on reasonable request.
