# Supplementary material for: Single‐cell and spatial transcriptomics uncover neoadjuvant chemotherapy‐resistant malignant cells with inhibitory signalling on B cells in gastric cancer
Source: Clin Transl Med. 2026 Feb 2;16(2):e70600. doi: 10.1002/ctm2.70600 (PMC12865221; doi:10.1002/ctm2.70600)
Supplement: Supplementary file 1 — Supporting information [file CTM2-16-e70600-s008.docx]

- **Supplementary Methods**

1. **Tumor sample collection**

We collected malignant tumor samples from five patients (**Supplementary Table S1**) who were initially diagnosed, underwent neoadjuvant therapy, and subsequently underwent surgical resection at Ruijin Hospital (Shanghai, China). The preoperative chemotherapy regimens included fluorouracil or its derivatives, such as capecitabine and tegafur, combined with oxaliplatin, with some patients additionally receiving docetaxel. The specific treatment plans were discussed and formulated by the Hospital's multidisciplinary gastric cancer consultation team, and the chemotherapy efficacy also evaluated by the team. The resultant data showed that, among these five patients, three exhibited the significant response to neoadjuvant therapy and the remaining two ones had no response to the therapy.

Post-surgical tumor samples were immediately collected following resection. The specific steps were as follows: (1) Tumor tissues were cut into approximately 200 mg pieces, ensuring that the gross tumor sample was preserved for pathological diagnosis; (2) The tissues were washed with PBS; (3) The tumor samples were placed in Miltenyi MACS Tissue Storage Solution and transported to the laboratory at 4°C.

1. **Whole-exome sequencing (WES) data analysis**

The resected five tumors from the above patients were obtained for WES analysis. Mutation annotation files (MAF) were generated using the human reference genome (GRCh38). Quality control (QC) measures were applied to ensure high-confidence mutation calls, including filtering out low-quality reads, removing artifacts, and ensuring adequate coverage across exomes. Mutation significance was assessed using MutSigCV1. This tool identifies significantly mutated genes by accounting for gene-specific background mutation rates and various covariates, such as gene expression levels and replication timing. Genes with a *q*-value below 0.05 were considered significantly mutated. The mutation signature analysis and waterfall visualization were performed using the maftools2 (version 2.20.0) package. Firstly, the trinucleotide context of each mutation was determined and the mutational profile was decomposed into known COSMIC signatures. Then, the proportion of each COSMIC signature contributing to the overall mutational landscape of each sample was quantified and the signature contributions were visualized using bubble plots.

1. **Bulk RNA-seq and differential expression analysis**

The bulk RNA-seq count data included five samples: tumor tissue from two non-responders and three responders. Gene IDs were converted from ENSEMBL identifiers to gene symbols using the org.Hs.eg.db database. Duplicate gene symbols were removed, keeping the entry with the highest average expression value. Differential expression analysis was performed using the DESeq2 (version 1.40.2) package3. A DESeqDataSet was created from the count data and sample metadata, with the experimental design specifying the comparison between responders and non-responders. The DESeq function was used to normalize the data and identify differentially expressed genes (DEGs). The results were correct for multiple testing using the Benjamini-Hochberg procedure. DEGs were defined based on |log2 fold change| > 1 and adjusted p-value < 0.05. Volcano plot was generated using the EnhancedVolcano (version 1.22.0) package.

1. **Single-cell isolation, library preparation and sequencing**

The tumor tissues were cut into approximately 1-2 mm³ fragments in RPMI-1640 medium (Gibco) containing 10% fetal bovine serum (FBS, Gibco) and enzymatically digested using a gentleMACS tissue processor (Miltenyi). The dissociated single cells were then stained with trypan blue to assess cell count and viability. Upon passing the assessment, the cells were loaded onto a 10X Chromium microfluidic chip to prepare single-cell RNA libraries. All subsequent steps were performed according to the manufacturer's standard protocol. The libraries were sequenced using an Illumina HiSeq-4000 sequencer.

1. **Preprocess, dimensionality reduction, clustering and cell type identification of single-cell sequencing data**

The droplet-based 10X single-cell RNA sequencing data were processed using the CellRanger software suite (version 3.0.0) provided by 10X Genomics. Gene expression levels were confirmed using the reference genome GRCh38. Quality control was first performed. Doublets were identified and removed using the scDblFinder package (version 1.17.3)^1^. Cells with a mitochondrial gene percentage above 30% were filtered out to avoid low-quality or dying cells. Following quality control, the data were normalized using the LogNormalize method with a scale factor of 10,000. Variable features were identified using the vst method, and data scaling was performed while regressing out the percentage of mitochondrial genes. Principal Component Analysis (PCA) was conducted on the highly variable genes, and the elbow plot was used to determine the optimal number of principal components to retain. Harmony (version 0.1.1) was applied to correct for batch effects^2^. After batch effect correction, Uniform Manifold Approximation and Projection (UMAP) was performed for dimensionality reduction, and the nearest neighbors were identified in the integrated dataset. Clustering was performed at resolution of 0.1 to identify cell populations. Markers for each cluster were identified using the *FindAllMarkers* function and cells were annotated based on known markers and markers collected in CellMarker 2.0^3^.

1. **Inference of genomic DNA copy number variations (CNV)**

We utilized the InferCNV package (version 1.8.1) to analyze CNVs in epithelial cells from our single-cell RNA sequencing dataset. Immune cells, including T cells, B cells, myeloid cells, and mast cells were subsetted from the overall scRNA-seq data and combined with the epithelial cells. Epithelial cells were labeled as the target cells, and immune cells served as the reference group. A subset of immune cells (labeled as 'spike_immune_cell') was randomly selected and inserted into the epithelial cells. The InferCNV analysis was run with the following parameters:cutoff: 0.1; clustering: Ward.D2 method without pre-clustering by cell groups; denoising: enabled; HMM: disabled. The hierarchical clustering dendrogram generated by InferCNV was processed to identify malignant and benign cell clusters according to the position of inserted immune cells.

1. **Pseudotime analysis**

We performed pseudotime analysis using the Monocle package (version 2.28.0)^4^. After creating a CellDataSet object, we estimated size factors and dispersions. Dimensionality reduction was performed using the DDRTree method, followed by cell ordering to infer pseudotime trajectories. Root state was determined according to the results of Vector and CytoTRACE^5,6^. Vector-based pseudotime inference was conducted to complement the Monocle analysis. Specifically, we constructed a grid and network to model the cell transitions and calculated Quantum Polarization scores. The Vector automatically identified center to determine the starting point for trajectory and visualized differentiation pathways through arrows above UMAP plot.

1. **Cell differentiation inference**

Cell differentiation potential was assessed using CytoTRACE (version 0.3.3), which estimates the differentiation state of cells based on gene expression data. The results were visualized in conjunction with the Monocle pseudotime trajectories.

1. **Functional enrichment and analysis**

Differentially expressed genes were identified and ranked by average log fold-change using *FindAllMarkers*. Top genes were subjected to Gene Ontology (GO) enrichment analysis using the clusterProfiler package (version 4.8.3) ^7^. Significant GO terms were visualized, highlighting key biological processes associated with each cell cluster. Down-regulated genes (DEGs with log2 fold change < -1) were converted to Entrez IDs for further analysis and the GO enrichment analysis was performed for biological processes (BP) specifically and the p-values were corrected using the Benjamini-Hochberg procedure as well. The top 15 enriched GO terms for down-regulated genes were identified based on adjusted p-values.

1. **Cell-cell communication analysis**

Cell-cell communication networks were analyzed using the CellChat package (version 2.1.2)^8^. We utilized a subset of the CellChatDB focusing on secreted signaling. Overexpressed genes and interactions were identified, followed by computation of communication probabilities and pathway probabilities.

1. **Gene set enrichment analysis (GSEA)**

Differentially expressed genes were identified using the *FindMarkers* function, with log fold-change thresholds set to 0. Gene lists were generated and mapped to ENTREZ IDs. The gene lists were sorted by log fold-change and used for GSEA using the clusterProfiler package. The GO gene sets were utilized for analysis, and results were corrected by Benjamini-Hochberg method.

1. **Transcription factor analysis**

To infer gene regulatory networks, we employed pySCENIC (version 0.12.1)^9^. Initially, the pySCENIC GRN step was used to identify co-expression modules between transcription factors (TFs) and genes from the provided expression matrix using the GRNBoost2 algorithm. Next, we performed TF-motif enrichment analysis using ctx step. This step involved ranking the databases and identifying direct targets of TFs through motif enrichment. Finally, the activity of each regulon in individual cells was assessed using the AUCell algorithm.

1. **Spatial transcriptome data acquisition and preprocessing**

Spatial transcriptome data were acquired from public GSE251950, a 10X Visium spatial transcriptomics dataset of primary gastric cancers, and preprocessed using the Seurat package (version 4.3.0)^10^. In details, tissue coordinates were obtained, and spots classified as "not on tissue" were filtered out. Mitochondrial and ribosomal genes were excluded from the analysis. Spots were retained based on quality metrics, including number of features and counts. Data normalization was performed using SCTransform and log normalization methods. Dimensionality reduction was achieved via PCA and UMAP, followed by clustering.

1. **Cell type decomposition**

Cell type decomposition was performed using the spacexr package (version 2.2.1). It broke down the RNA sequencing mixture into individual cell types, enabling the assignment of cell types to spatial transcriptome pixels. In this study, single-cell RNA-seq data annotated in 2.4 were used to construct a reference dataset. The resulting weights from the decomposition were then integrated into the spatial transcriptome dataset.

1. **Cell colocalization analysis**

Colocalization analysis was conducted using the MistyR package (version 1.8.1)^11^. Spatial views were defined for intra-, juxta-, and para-spot contexts, with corresponding assays and features. MistyR was then applied to run the colocalization analysis, and the results were collected for further interpretation. MistyR captured cell type-specific relationships between different anatomical regions.

1. **Homotypic and heterotypic cell network analysis**

Nearest neighbor analysis was performed using the *dbscan::kNN* function to identify the six nearest neighbors for each spot. Distances and weights for these connections were calculated. The spatial network was constructed by retaining edges with a distance less than 200 units or ranked within the top four nearest neighbors. For each cell type of interest in the spatial network, we selected spots with a deconvolution score above a specified threshold. We then initialized a degree matrix to store the degree of each spot, which represents the number of neighboring spots with the same cell type above the threshold. The degree of each spot was calculated by summing the scores of its neighboring spots.

For heterotypic cell network analysis, two different cell types were selected based on respective thresholds. The network connections between spots containing different cell types were identified. An enrichment score for the heterotypic cell network was calculated by determining the number of neighboring spots containing the second cell type for each spot containing the first cell type. This method provides a comprehensive approach to analyze and visualize the spatial distribution and network interactions of specific cell types within tissue sections.

Moreover, we used COMMunication analysis by Optimal Transport (COMMOT) (version 0.0.3) to screen intercellular communication in the spatial transcriptome^12^. A ligand-receptor database for human secreted signaling pathways was retrieved from the CellTalker library with ‘ct.pp.ligand_receptor_database’, specifying the CellChat database. For each selected ligand-receptor pair, spatial communication was inferred using the ‘ct.tl.spatial_communication’ function. The distance threshold for spatial interaction was set to 500 μm. The results of the spatial communication analysis were visualized to identify sending and receiving cells for the selected pairs. Directional communication was analyzed using the ‘ct.tl.communication_direction’ function, setting a nearest-neighbor parameter k = 5.

1. **Immunohistochemistry**

Formalin-fixed, paraffin-embedded (FFPE) tissue blocks of GC patients were sectioned for immunohistochemical staining. Slides were deparaffinized, rehydrated, and subjected to high-pressure for antigen retrieval in EDTA buffer. The endogenous peroxidase activity was then blocked by incubating the slides in 3% hydrogen peroxide. Then, slides were blocked in 5% BSA for 1h and incubated with primary antibodies (pan-CK, Afantibody, AF20164; SPP1, R&D, 22952-1-AP; CD19, Servicebio, GB11061; CD44, Biolegend, 103001) overnight at 4°C. After washing, secondary antibodies were incubated for 1h, followed by DAPI (Thermo Fisher, D1306) staining. Finally, slides were scanned using 3DHISTECH digital slide scanner.

1. **Survival analysis**

Gene expression data of GC patients were extracted from the TCGA-STAD dataset (https://www.cancer.gov/ccg/research/genome-sequencing/tcga). Survival analysis was performed to evaluate the prognostic significance of specific genes in the dataset. Clinical data, including overall survival (OS) time and status, was merged with gene expression data to form the final dataset for survival analysis. Optimal cut-off points for gene expression were determined using the *surv_cutpoint* function from the survminer package (version 0.4.9). Samples were categorized into high and low gene expression groups based on corresponding cut-off. Kaplan-Meier survival curves were generated for each gene using the survival package (version 3.5-7). Log-rank test was used to compare survival distributions between groups.

1. **Cell lines**

MFC cells were cultured in complete RPMI-1640 medium containing 10% fetal bovine serum (FBS). Cells were grown at 37°C in a 5% CO2 setting.

1. **Lentiviral transfection and reagents**

Stable overexpression of SPP1 was successfully achieved by infecting OE-SPP1 lentiviral particles into MFC cells (Qingke Biology (Beijing, China)), after the cells continued to incubate for 48 h, the transfection rate was detected by fluorescence observation under the microscope, and then the stable strains were screened by continuing the incubation in medium containing 6ug/ml puromycin for 7 days.

1. **Western Blot**

Total protein was extracted using RIPA buffer containing a Protease Inhibitor Cocktail (Thermo Fisher) after washing cells with cold PBS. Lysates were clarified by centrifugation (12,000 ×g, 4°C, 10 min), and protein levels were quantified via the BCA method (Beyotime). Equal amounts of protein were resolved by SDS-PAGE using FuturePAGE 4–20% gels (ACE Biotechnology) and transferred to 0.2 μm NC membranes (Cytiva). Following blocking with 5% non-fat milk (1 h, room temperature), membranes were incubated overnight at 4°C with the primary antibody (22952-1-AP, Proteintech). Blots were then washed and incubated with secondary antibodies for 1 h at room temperature before imaging with an ImageQuant LAS 4000 mini (Cytiva).

1. **B cell culture in vitro**

For in vitro experiments, B cells were isolated from splenic single-cell suspensions utilizing a STEMCELL isolation kit. The cells were resuspended in complete RPMI-1640 medium (10% FBS) with or without 20% MFC tumor cell supernatant containing 10 μg/mL LPS, which served to maintain cellular activity. Experimental groups were kept at 37°C in a 5% CO₂ incubator, with harvest points at 24 h, 48 h, or other indicated times.

1. **Coculture of B cells and MFC tumor cells**

B cells were purified from spleen using B cell isolation kit (STEMCELL). SPP1-OE or SPP1-vehicle MFC tumor cells (5∗103) were coated onto the 48-well plate. The purified B cells (5∗105 cells/well) were cultured in the presence of LPS (10μg/mL) for 48 h. Activated B cells were assessed by flow cytometry.

1. **Flow cytometry**

Cells were resuspended in staining buffer (PBS, 1% BSA, 0.05% NaN₃) and incubated with specific fluorochrome-conjugated antibodies and 1 µg anti-CD16/CD32 (Thermo Fisher) for 30 min at 4°C. After washing three times, cells were analyzed on a CyAn ADP Analyzer (Beckman, Coulter, Pasadena, CA, USA). Data processing was carried out using FlowJo software (Tree Star, Ashland, OR, USA).

- **Supplementary Discussion**

Our study investigated the treatment responsiveness of GC patients undergoing neoadjuvant therapy using scRNA-seq data. We observed significant differences in the tumor microenvironment (TME) between responders and non-responders, shedding light on potential molecular and cellular mechanisms of therapy resistance. Notably, non-responders exhibited the higher proportions of epithelial cells and fibroblasts but a lower proportion of B cells compared to responders.

Fibroblasts are known to remodel the extracellular matrix and promote immune evasion, which may contribute to the reduced responsiveness observed in non-responders^13^. Our study investigated the treatment responsiveness of GC patients undergoing neoadjuvant therapy using scRNA-seq data. We observed significant differences in the tumor microenvironment (TME) between responders and non-responders, shedding light on potential molecular and cellular mechanisms of therapy resistance. Notably, non-responders exhibited the higher proportions of epithelial cells and fibroblasts but a lower proportion of B cells compared to responders. Fibroblasts are known to remodel the extracellular matrix and promote immune evasion, which may contribute to the reduced responsiveness observed in non-responders^13^. In addition, recent studies have explored how stromal components collectively shape the macro-landscape of immune evasion^14^. Complementarily, Zhang et al. summarized recent clinical advancements and the persistent challenges of therapeutic resistance facing PD-1/PD-L1 inhibitors and emerging modalities^15^. Here, we focus on the reduced proportion of B cells in non-responders.

B cell lineages not only play an important role in the initiation and progression of tumors, but also are related to the reactivity of chemotherapy. Some studies have shown that patients with muscle-invasive bladder cancer exhibiting higher CD19^+^ B cell infiltration were more sensitive to platinum-based chemotherapy^16^. In addition, neoadjuvant chemotherapy increased cytotoxic T cells, tissue-resident memory T cells, and B cell infiltration in resectable non-small cell lung cancer^17^. Furthermore, chemotherapy also affects the number and function of B cells within TME, thereby modulating anti-tumor immunity and therapeutic efficacy. Most studies have shown that chemotherapy causes a decrease in the number of B cells^18,19^. However, some reports showed no significant change or even an increase in B-cell infiltration^17,20^. In addition, chemotherapy alters the proportion of B cell subsets, often increasing naive B cells but decreasing memory B cells^21^.

The tumor cells exhibit more essential features, and analyzing their heterogeneity may be helpful to the prediction of a patient's response to neoadjuvant therapy. In this study, a distinct subpopulation of malignant epithelial cells (EPI5) was predominant in remnant tumor samples from non-responders. These cells displayed strong anti-apoptotic properties and an undifferentiated state, contributing to their high malignancy and drug resistance. The reduced cell-cell adhesion observed in EPI5 cells may further facilitate tumor progression and metastasis. Moreover, the high expression of EPI5 markers (HSPA1B, PLA2G2A and RAMP1) was positively correlated with poor prognosis, highlighting the negative role of EPI5 in therapy resistance and poor patient outcomes. While Li et al. previously identified an EPI5 cluster linked to p53 and EMT pathways in cervical cancer^22^, our study uniquely characterizes the EPI5 subpopulation in gastric cancer. We define these cells by their robust anti-apoptotic properties and reduced cell-cell adhesion, establishing a direct link between this specific phenotype and intrinsic resistance to neoadjuvant chemotherapy. This is consistent with previous studies, which have shown that HSPA1B is highly expressed in lymphocytes of patients with advanced hepatocellular carcinoma and is associated with poor prognosis^23^. *PLA2G2A* encodes phospholipase A2 IIA group, which belongs to secretory PLA2 (sPLA2) family. It exhibited high expression and enzyme activity to promote cancer progression through hydrolyzing cellular phospholipids and releasing arachidonic acid (AA) and lysophosphatidic acid (LPA)^24,25^. AA can be further metabolized into prostaglandins and leukotrienes, and can promote cell proliferation and tumor progression^26^. *RAMP1* encodes a receptor (calcitonin) active modification protein that is required to transport calcitonin receptor-like receptors (CRLR) to the plasma membrane. Many studies have shown that RAMP1 is a poor prognostic marker for multiple carcinomas, and targeting the CALCB/RAMP1 axis can inhibit the growth of Ewing's sarcoma^27,28^. Therefore, we propose that targeting EPI5 cells could enhance therapeutic efficacy and improve prognosis in GC patients.

Interestingly, our cell-cell communication analysis revealed the extensive interactions between malignant epithelial cells and B cells, which were mediated primarily by the immunosuppressive SPP1 signaling pathway. SPP1, also named as osteopontin, is a multifunctional extracellular matrix protein involved in various biological processes, including cell adhesion, migration, and signal transduction^29^. The overexpression of SPP1 has been strongly associated with increased tumor invasiveness and metastatic potential in multiple cancer type^30-32^.It has been known that SPP1 is expressed in a limited number of cell types, such as fibroblasts, macrophages, dendritic cells, and lymphocytes. Additionally, SPP1 is also expressed by cancer cells. Previous studies have shown a correlation between increased SPP1 expression on tumor cells and poor prognosis for many cancer types, where the SPP1-CD44 axis has been implicated in contributing to chemotherapy resistance in solid cancers. However, distinct from previous findings where CAF-derived SPP1 enhances tumor stemness or hypoxia-induced SPP1 impairs dendritic cell activation^31,33^, our research identifies a novel mechanism in the GC microenvironment. We reveal that the SPP1-CD44 axis acts as a specific mediator of crosstalk between chemo-resistant malignant epithelial cells and B cells, facilitating an immunosuppressive niche through direct tumor-B cell interaction. Significantly, in this study, EPI5 cells emerged as primary senders of the SPP1 signal, which was received by B cells. The robust SPP1 signaling suggests an underlying mechanism by which EPI5 cells suppress B cell activity, thereby contributing to the formation of an immunosuppressive TME. Furthermore, spatial transcriptomics analysis also provided compelling evidence of colocalization and interaction between EPI5 cells and activated B cells within tumor tissues. This spatial arrangement substantiates the SPP1-mediated immunosuppressive effects of EPI5 on B cells, consequently reinforcing their role in therapy resistance.

Our findings emphasize the importance of targeting anti-apoptotic undifferentiated epithelial cells, as well as the interaction between EPI5 cells and B cells to overcome neoadjuvant therapy resistance in GC patients. Strategies aimed at inhibiting SPP1 signaling or modulating interactions between EPI5 and B cells could enhance treatment efficacy. Further investigation into the molecular mechanisms underlying the anti-apoptotic and immunosuppressive properties of undifferentiated epithelial cells may uncover novel therapeutic targets.

In summary, this study provides comprehensive insights into the cellular dynamics and interactions within the TME of GC patients undergoing neoadjuvant therapy, paving the way for developing more effective treatment strategies aimed at improving patient outcomes.

- **References**

1 Germain, P. L., Lun, A., Garcia Meixide, C., Macnair, W. & Robinson, M. D. Doublet identification in single-cell sequencing data using scDblFinder. *F1000Res* **10**, 979, doi:10.12688/f1000research.73600.2 (2021).

2 Korsunsky, I. *et al.* Fast, sensitive and accurate integration of single-cell data with Harmony. *Nat Methods* **16**, 1289-1296, doi:10.1038/s41592-019-0619-0 (2019).

3 Hu, C. *et al.* CellMarker 2.0: an updated database of manually curated cell markers in human/mouse and web tools based on scRNA-seq data. *Nucleic Acids Res* **51**, D870-d876, doi:10.1093/nar/gkac947 (2023).

4 Trapnell, C. *et al.* The dynamics and regulators of cell fate decisions are revealed by pseudotemporal ordering of single cells. *Nat Biotechnol* **32**, 381-386, doi:10.1038/nbt.2859 (2014).

5 Zhang, F., Li, X. & Tian, W. Unsupervised Inference of Developmental Directions for Single Cells Using VECTOR. *Cell Rep* **32**, 108069, doi:10.1016/j.celrep.2020.108069 (2020).

6 Gulati, G. S. *et al.* Single-cell transcriptional diversity is a hallmark of developmental potential. *Science* **367**, 405-411, doi:10.1126/science.aax0249 (2020).

7 Wu, T. *et al.* clusterProfiler 4.0: A universal enrichment tool for interpreting omics data. *Innovation (Camb)* **2**, 100141, doi:10.1016/j.xinn.2021.100141 (2021).

8 Jin, S. *et al.* Inference and analysis of cell-cell communication using CellChat. *Nat Commun* **12**, 1088, doi:10.1038/s41467-021-21246-9 (2021).

9 Aibar, S. *et al.* SCENIC: single-cell regulatory network inference and clustering. *Nat Methods* **14**, 1083-1086, doi:10.1038/nmeth.4463 (2017).

10 Butler, A., Hoffman, P., Smibert, P., Papalexi, E. & Satija, R. Integrating single-cell transcriptomic data across different conditions, technologies, and species. *Nat Biotechnol* **36**, 411-420, doi:10.1038/nbt.4096 (2018).

11 Tanevski, J., Flores, R. O. R., Gabor, A., Schapiro, D. & Saez-Rodriguez, J. Explainable multiview framework for dissecting spatial relationships from highly multiplexed data. *Genome Biol* **23**, 97, doi:10.1186/s13059-022-02663-5 (2022).

12 Cang, Z. *et al.* Screening cell-cell communication in spatial transcriptomics via collective optimal transport. *Nat Methods* **20**, 218-228, doi:10.1038/s41592-022-01728-4 (2023).

13 Li, C., Teixeira, A. F., Zhu, H. J. & Ten Dijke, P. Cancer associated-fibroblast-derived exosomes in cancer progression. *Mol Cancer* **20**, 154, doi:10.1186/s12943-021-01463-y (2021).

14 Yasuda, T. & Wang, Y. A. Gastric cancer immunosuppressive microenvironment heterogeneity: implications for therapy development. *Trends Cancer* **10**, 627-642, doi:10.1016/j.trecan.2024.03.008 (2024).

15 Zhang, P. *et al.* Immunotherapy for gastric cancer: Advances and challenges. *MedComm – Oncology* **3**, e92, doi:<https://doi.org/10.1002/mog2.92> (2024).

16 Jiang, Q. *et al.* CD19(+) tumor-infiltrating B-cells prime CD4(+) T-cell immunity and predict platinum-based chemotherapy efficacy in muscle-invasive bladder cancer. *Cancer Immunol Immunother* **68**, 45-56, doi:10.1007/s00262-018-2250-9 (2019).

17 Gaudreau, P. O. *et al.* Neoadjuvant Chemotherapy Increases Cytotoxic T Cell, Tissue Resident Memory T Cell, and B Cell Infiltration in Resectable NSCLC. *J Thorac Oncol* **16**, 127-139, doi:10.1016/j.jtho.2020.09.027 (2021).

18 Shang, X., Zhang, C., Wang, K. & Wang, H. Neoadjuvant chemotherapy remodels the tumor immune microenvironment by increasing activated and cytotoxic T cell, decreasing B cells and macrophages in small cell lung cancer. *J Transl Med* **21**, 645, doi:10.1186/s12967-023-04526-4 (2023).

19 Froidevaux, S. & Loor, F. Myeloid and lymphoid cell alterations in normal mice exposed to chemotherapy with doxorubicin and/or the multidrug-resistance reversing agent SDZ PSC 833. *Int J Cancer* **59**, 133-140, doi:10.1002/ijc.2910590123 (1994).

20 Montfort, A. *et al.* A Strong B-cell Response Is Part of the Immune Landscape in Human High-Grade Serous Ovarian Metastases. *Clin Cancer Res* **23**, 250-262, doi:10.1158/1078-0432.Ccr-16-0081 (2017).

21 Li, Z. *et al.* B-cell performance in chemotherapy: Unravelling the mystery of B-cell therapeutic potential. *Clin Transl Med* **14**, e1761, doi:10.1002/ctm2.1761 (2024).

22 Li, X. *et al.* Single-cell RNA-sequencing dissects cellular heterogeneity and identifies two tumor-suppressing immune cell subclusters in HPV-related cervical adenosquamous carcinoma. *J Med Virol* **94**, 6047-6059, doi:10.1002/jmv.28084 (2022).

23 He, Y. *et al.* Single-cell profiling of human CD127(+) innate lymphoid cells reveals diverse immune phenotypes in hepatocellular carcinoma. *Hepatology* **76**, 1013-1029, doi:10.1002/hep.32444 (2022).

24 Peng, Z., Chang, Y., Fan, J., Ji, W. & Su, C. Phospholipase A2 superfamily in cancer. *Cancer Lett* **497**, 165-177, doi:10.1016/j.canlet.2020.10.021 (2021).

25 Brglez, V., Lambeau, G. & Petan, T. Secreted phospholipases A2 in cancer: diverse mechanisms of action. *Biochimie* **107 Pt A**, 114-123, doi:10.1016/j.biochi.2014.09.023 (2014).

26 Khan, S. A. & Ilies, M. A. The Phospholipase A2 Superfamily: Structure, Isozymes, Catalysis, Physiologic and Pathologic Roles. *Int J Mol Sci* **24**, doi:10.3390/ijms24021353 (2023).

27 Xie, L., Xiao, W., Fang, H. & Liu, G. RAMP1 as a novel prognostic biomarker in pan-cancer and osteosarcoma. *PLoS One* **18**, e0292452, doi:10.1371/journal.pone.0292452 (2023).

28 Dallmayer, M. *et al.* Targeting the CALCB/RAMP1 axis inhibits growth of Ewing sarcoma. *Cell Death Dis* **10**, 116, doi:10.1038/s41419-019-1372-0 (2019).

29 Rangaswami, H., Bulbule, A. & Kundu, G. C. Osteopontin: role in cell signaling and cancer progression. *Trends Cell Biol* **16**, 79-87, doi:10.1016/j.tcb.2005.12.005 (2006).

30 Qi, J. *et al.* Single-cell and spatial analysis reveal interaction of FAP(+) fibroblasts and SPP1(+) macrophages in colorectal cancer. *Nat Commun* **13**, 1742, doi:10.1038/s41467-022-29366-6 (2022).

31 Nallasamy, P. *et al.* Pancreatic Tumor Microenvironment Factor Promotes Cancer Stemness via SPP1-CD44 Axis. *Gastroenterology* **161**, 1998-2013.e1997, doi:10.1053/j.gastro.2021.08.023 (2021).

32 Eun, J. W. *et al.* Cancer-associated fibroblast-derived secreted phosphoprotein 1 contributes to resistance of hepatocellular carcinoma to sorafenib and lenvatinib. *Cancer Commun (Lond)* **43**, 455-479, doi:10.1002/cac2.12414 (2023).

33 Chen, S. *et al.* Hypoxia-driven tumor stromal remodeling and immunosuppressive microenvironment in scirrhous HCC. *Hepatology* **79**, 780-797, doi:10.1097/hep.0000000000000599 (2024).
