# Supplementary figures and images for: Single‐cell and spatial transcriptomics uncover neoadjuvant chemotherapy‐resistant malignant cells with inhibitory signalling on B cells in gastric cancer
Source: Clin Transl Med. 2026 Feb 2;16(2):e70600. doi: 10.1002/ctm2.70600 (PMC12865221; doi:10.1002/ctm2.70600)

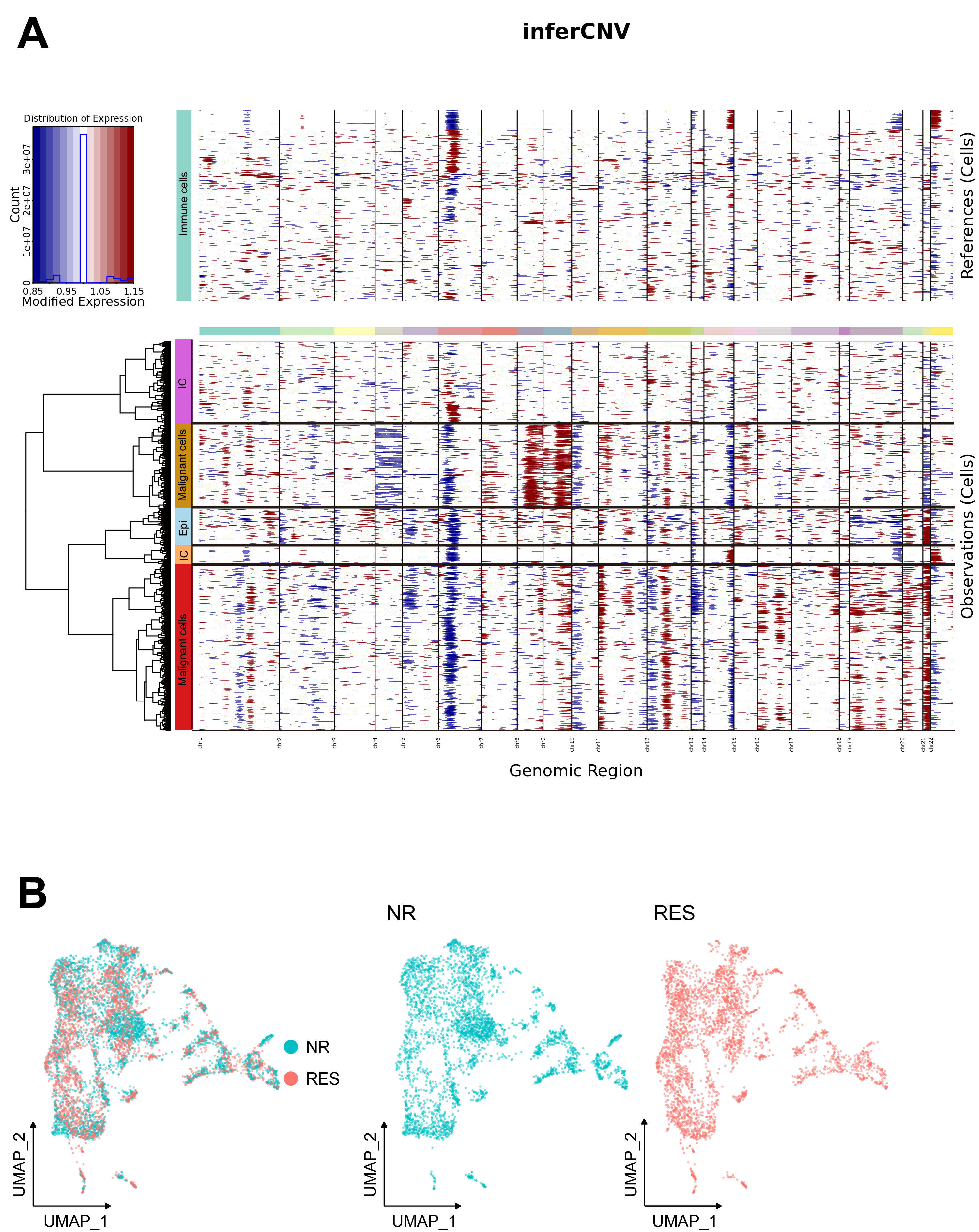

Supplement: Supplementary file 2 — Supporting information [file CTM2-16-e70600-s007.tif]

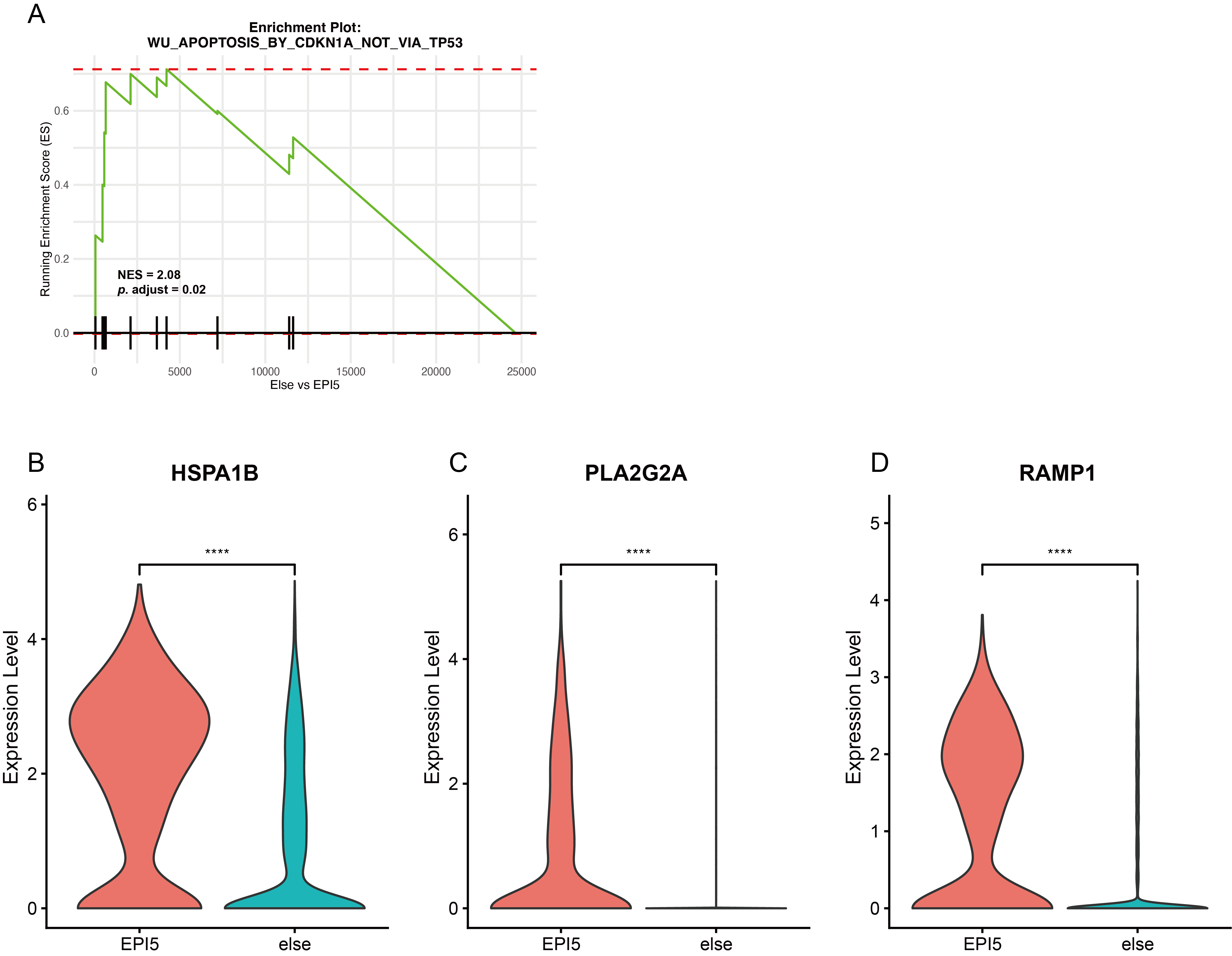

Supplement: Supplementary file 3 — Supporting information [file CTM2-16-e70600-s005.tif]

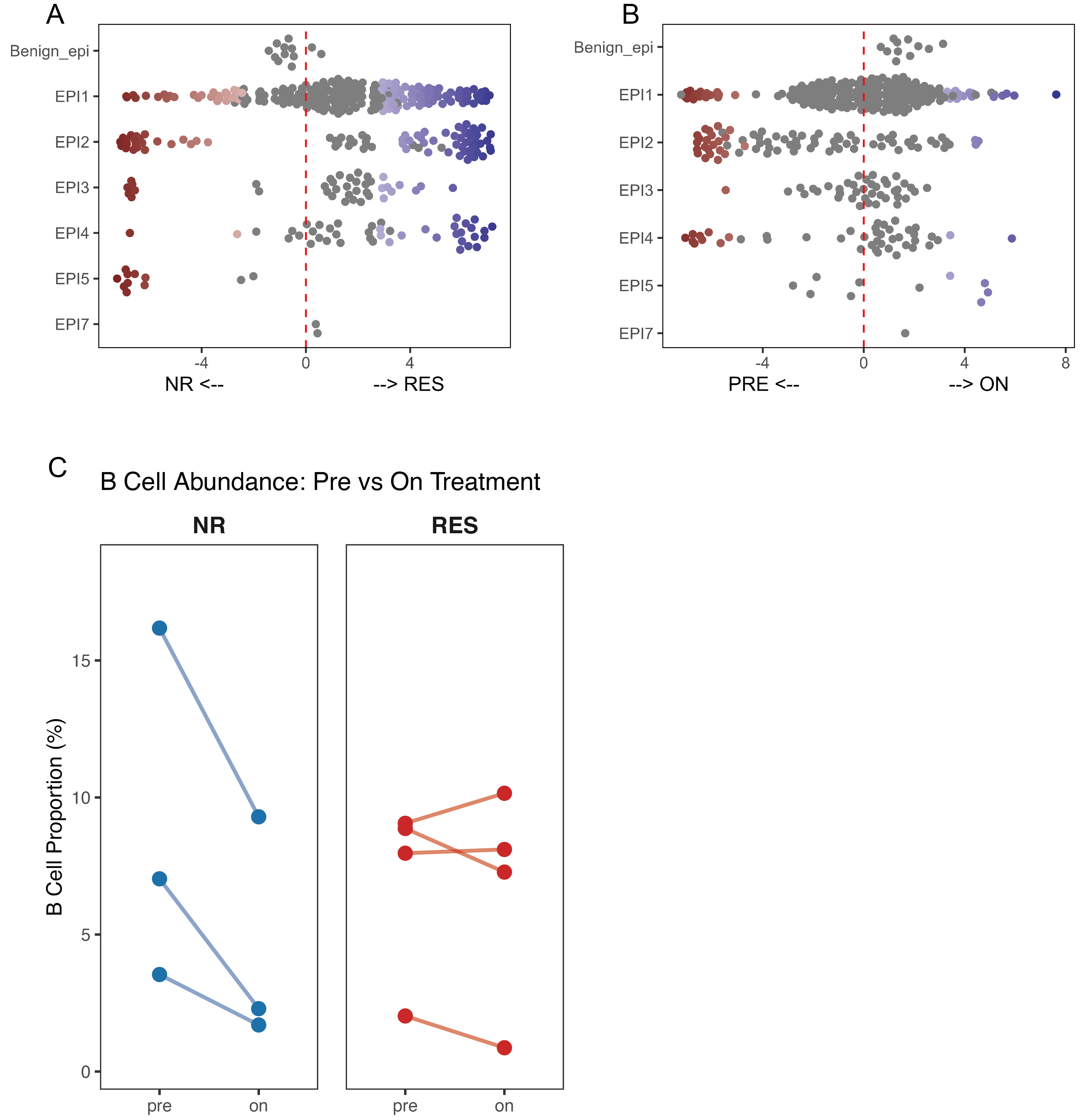

Supplement: Supplementary file 4 — Supporting information [file CTM2-16-e70600-s004.tif]

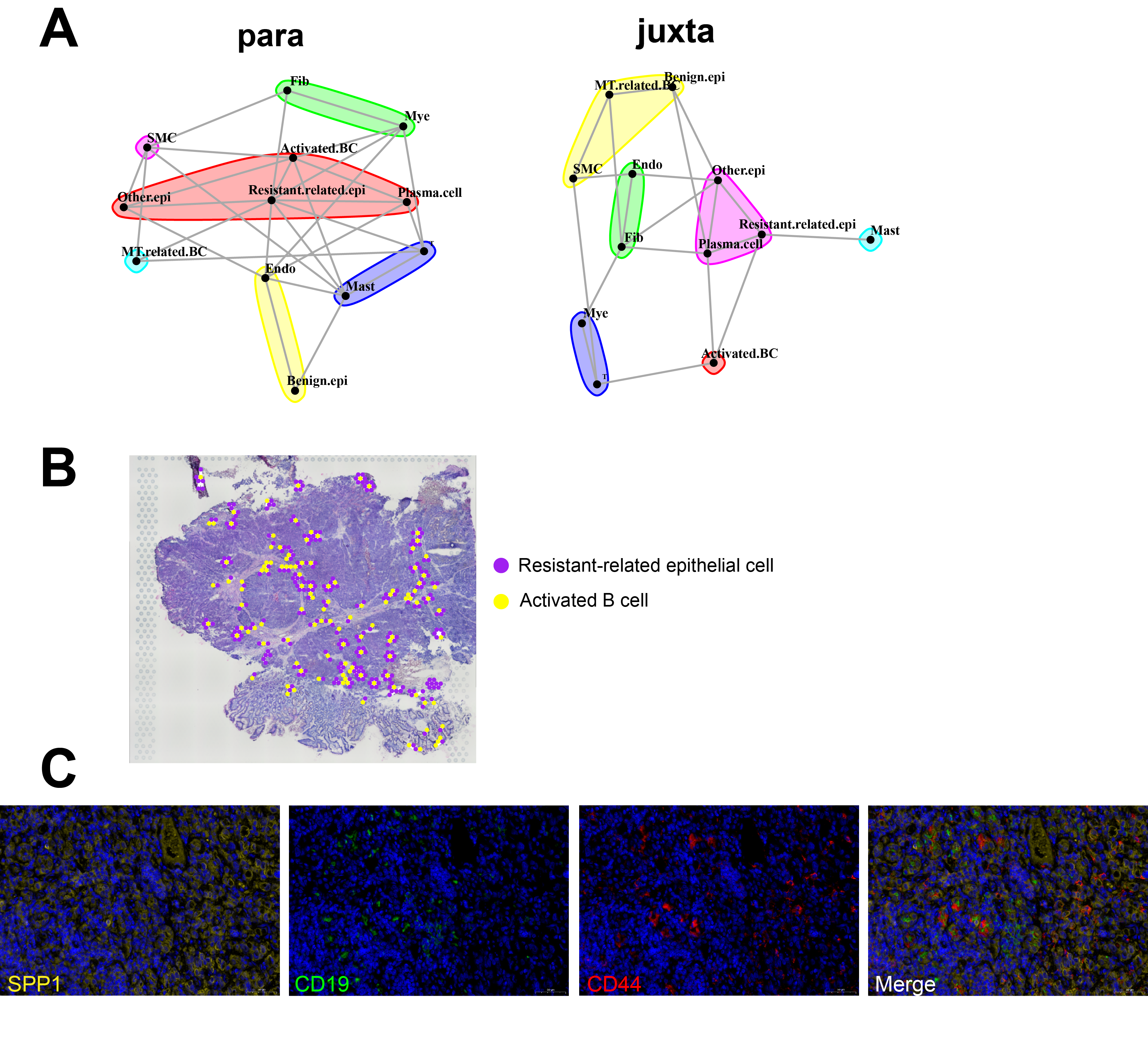

Supplement: Supplementary file 5 — Supporting information [file CTM2-16-e70600-s003.tif]
