# Supplementary material for: Single‐cell and spatial transcriptomics uncover neoadjuvant chemotherapy‐resistant malignant cells with inhibitory signalling on B cells in gastric cancer
Source: Clin Transl Med. 2026 Feb 2;16(2):e70600. doi: 10.1002/ctm2.70600 (PMC12865221; doi:10.1002/ctm2.70600)
Supplement: Supplementary file 6 — Supporting information [file CTM2-16-e70600-s002.docx]

**Supplementary figure legend**

**Supplementary Figure 1. Identification of malignant cells through the analysis of genomic DNA copy number variations (CNVs). (A)** Genomic DNA copy number variation (CNV) alterations inferred by the InferCNV software across different chromosomes. **(B)** UMAP plot showing epithelial cells grouped by neoadjuvant therapy response.

**Supplementary Figure 2 Spatial transcriptome analysis of gastric cancer. (A)** Gene set enrichment analysis (GSEA) of the CDKN1A-mediated survival pathway. **(B-D)** Expression level of HSPA1B, PLA2G2A and RAMP1 in EPI5 and other EPI clusters.

**Supplementary Figure 3 Comparative analysis of cell type abundance in responders and non-responders pre- and post-neoadjuvant chemotherapy. (A)** Beeswarm plots illustrating the abundance of distinct EPI clusters in responders versus non-responders. **(B)** Beeswarm plots displaying the abundance of EPI clusters pre- and post-neoadjuvant chemotherapy. **(C)** Box plots comparing the relative abundance of B cells between responders and non-responders at baseline and post-treatment.

**Supplementary Figure 4 Spatial transcriptome and immunofluorescence analysis of gastric cancer. (A)** Paracrine (para) and juxta networks inferred by MistyR algorithm. **(B)** The colored spots indicate resistant-related epithelial cells (purple) and surrounding activated B cells (yellow). **(C)** Typical image showing SPP1-positive epithelial cells and CD44-positive B cells of GC tissues after neoadjuvant chemotherapy, to achieve more precise cellular localization as determined by IF staining.
