# Supplementary material for: Single‐cell and spatial transcriptomics uncover neoadjuvant chemotherapy‐resistant malignant cells with inhibitory signalling on B cells in gastric cancer
Source: Clin Transl Med. 2026 Feb 2;16(2):e70600. doi: 10.1002/ctm2.70600 (PMC12865221; doi:10.1002/ctm2.70600)
Supplement: Supplementary file 7 — Supporting information [file CTM2-16-e70600-s001.docx]

| **Table S1. Clinical Characteristics of 5 GC Patients Involved in This Study** | | | |  |  |  |
| --- | --- | --- | --- | --- | --- | --- |
| Platform | Characteristics | GC9 | GC11 | GC12 | GC14 | GC15 |
| 10x Genomics | Age |  |  |  |  |  |
|  | Gender |  |  |  |  |  |
|  | Histological type^a^ | ADC | ADC | ADC | ADC | ADC |
|  |  |  |  |  |  |  |
|  | Location | Cardia | Body | Cardia | Cardia | Antrum |
|  | pTNM: T | 3 | 4 | 3 | 3 | 3 |
|  | pTNM: N | 0 | 2 | 3 | 1 | 0 |
|  | pTNM: M | 0 | 0 | 0 | 0 | 0 |
|  | Grade | Low- | Low or moderate | Low- | Low or moderate | Low- |
|  |  | differentiated | differentiated | differentiated | differentiated | differentiated |
|  | MMR status^b^ | pMMR | pMMR | pMMR | pMMR | dMMR |
|  | Regimens^c^ | DOS | DOS | SOX | DOS | XELOX |
|  | Cycle | 4 | 4 | 4 | 3 | 4 |
|  | Outcome^d^ | PR | PR | PR | SD | PD |

^a^ADC, adenocarcinoma.

^b^pMMR, MMR-proficient; dMMR, MMR deficient.

^c^DOS, Docetaxel+Oxaliplatin+ tegafur/gimeracil/oteracil potassium; SOX, tegafur/gimeracil/oteracil potassium+Oxaliplatin; XELOX, capecitabine+ Oxaliplatin.

^d^PR, partial response; SD, stable disease; PD, progressive disease.
