# Supplementary material for: Single‐cell and spatial transcriptomics uncover neoadjuvant chemotherapy‐resistant malignant cells with inhibitory signalling on B cells in gastric cancer
Source: Clin Transl Med. 2026 Feb 2;16(2):e70600. doi: 10.1002/ctm2.70600 (PMC12865221; doi:10.1002/ctm2.70600)
Supplement: Supplementary file 8 — Supporting information [file CTM2-16-e70600-s006.docx]

| **Table S2. Clinical Characteristics of GC Patients Involved in This Study** | | | |  |  |  |  |
| --- | --- | --- | --- | --- | --- | --- | --- |
|  | Histological type^a^ | Location | pTNM | MMR status^b^ | Regimens^c^ | Cycle | Outcome^d^ |
| 1 | ADC | Cardia | T4N1M0 | pMMR | XELOX | 4 | PR |
| 2 | ADC | Cardia | T3N3M0 | pMMR | DOS | 7 | PR |
| 3 | ADC | Body | T3N2M0 | dMMR | XELOX | 6 | PR |
| 4 | ADC | Antrum | T4N3M0 | dMMR | FLOT | 4 | PR |
| 5 | ADC | Cardia | T3N2M0 | dMMR | SOX | 8 | PR |
| 6 | ADC | Antrum | T3N3M0 | pMMR | DOS | 3 | PD |
| 7 | ADC | Body | T3N3M0 | pMMR | SOX | 4 | PD |
| 8 | ADC | Cardia | T4N3M0 | dMMR | DOS | 8 | PD |
| 9 | ADC | Body | T4N1M0 | dMMR | FLOT | 8 | PD |

^a^ADC, adenocarcinoma.

^b^pMMR, MMR-proficient; dMMR, MMR deficient.

^c^DOS, Docetaxel+Oxaliplatin+ tegafur/gimeracil/oteracil potassium; SOX, tegafur/gimeracil/oteracil potassium+Oxaliplatin; XELOX, capecitabine+ Oxaliplatin. ;FLOT, fluorouracil+ leucovorin+ oxaliplatin+ docetaxel

^d^PR, partial response; SD, stable disease; PD, progressive disease.
